# Insights Into Gut Microbiota and Metabolic Alterations in Type 1 Diabetes Mellitus

**DOI:** 10.1002/dmrr.70211

**Published:** 2026-07-27

**Authors:** Caroline Fogagnolo, Gabriela Ueta Ortiz, Adelino Sanchez Ramos da Silva, Ellen Cristini de Freitas

**Affiliations:** ^1^ Ribeirao Preto Medical School University of Sao Paulo (USP) Ribeirao Preto Sao Paulo Brazil; ^2^ School of Physical Education and Sport of Ribeirao Preto University of Sao Paulo (USP) Ribeirao Preto Sao Paulo Brazil

Type 1 diabetes mellitus (T1DM) is a chronic autoimmune disease marked by immune responses against pancreatic β‐cell antigens, activating autoreactive T and B cells and leading to progressive β‐cell destruction, impaired insulin secretion, and hyperglycemia [1]. In T1DM, evidence suggests a synergistic interaction between altered gut microbiota, increased intestinal permeability, and immune dysregulation, contributing to loss of immune tolerance and β‐cell destruction [1].

Chronic hyperglycemia and low‐grade inflammatory profile in T1DM individuals may impair intestinal barrier function, increasing permeability [2], promoting immune activation and perpetuating β‐cell autoimmunity [3]. This supports a bidirectional relationship in which T1DM‐related immune and metabolic changes disrupt gut microbiota homoeostasis, while dysbiosis further amplifies immune dysfunction and disease severity.

In this context, we read with great interest the cross‐sectional study conducted by Li et al. [4] published in August 2025 in *Diabetes/Metabolism Research and Reviews* (41:6). The study examined the relationship between gut microbiota and T1DM duration, suggesting duration‐specific microbiome alterations. In long‐standing T1DM, microbial shifts reflect functional imbalance rather than isolated taxonomic changes, marked by reduced butyrate‐ and short‐chain fatty acid (SCFA)‐producing taxa and broader alterations in metabolically relevant bacteria. Notably, depletion of SCFA producers, including *Faecalibacterium*, *Fusicatenibacter*, *Lachnospira*, and *Subdoligranulum*, was consistently associated with poorer glycaemic control.

SCFAs play a central role in modulating the immune response, particularly through the regulation of B‐cell function and metabolism [5]. By attenuating excessive immune activation while promoting metabolic reprogramming towards plasma cell differentiation via pathways such as glycolysis, SCFAs favour more controlled and tolerogenic humoral responses [6]. Plasma cells, the terminally differentiated form of B cells, are fundamental to humoral immunity due to their capacity for sustained antibody secretion and immunological memory [7].

SCFAs also regulate cellular function through the inhibition of histone deacetylases (HDACs), leading to increased histone acetylation, which plays a key role in gene transcription. Among SCFAs, butyrate is the most potent HDAC inhibitor [8]. These epigenetic mechanisms have been associated with the regulation of essential metabolic pathways, including glycolysis and mitochondrial oxidative phosphorylation, critical for plasma cell differentiation and function [5]. Consequently, increased immunoglobulin production, particularly IgG and IgA, enhances immune protection. Moreover, SCFAs promote the expansion and activity of regulatory B cells, which secrete anti‐inflammatory cytokines such as interleukin‐10 (IL‐10), interleukin‐35 (IL‐35), and transforming growth factor beta 1 (TGF‐β1), reinforcing their role in immune modulation [9].

A key unresolved question is whether the decline in SCFA‐producing bacteria in long‐standing T1DM is a cause of disease progression or a consequence of chronic hyperglycemia and metabolic disturbances. Importantly, the cross‐sectional design of Li et al. [4] precludes causal inference, limiting interpretation to associations with disease duration. Evidence from longitudinal paediatric cohorts supports a potential modulatory role of the gut microbiota in T1DM development [10]. In genetically predisposed children, disease progression has been primarily observed in those whose gut microbiota becomes enriched with *Bacteroides dorei*, whereas at‐risk individuals lacking this microbial signature do not progress to overt disease. These findings suggest that genetic susceptibility alone may be insufficient, highlighting the potential contribution of specific microbial signatures to the initiation of autoimmunity.

Regarding mainly changes in serum metabolites, Li et al. [4] reported disturbances in taurine and hypotaurine metabolism, and vitamin B6 metabolism in these groups. Taurine (2‐aminoethanesulfonic acid) is a nitrogen‐based amino acid characterised by a sulfonic acid group instead of the standard carboxylic acid present in most amino acids [11]. Individuals with T1DM exhibit lower taurine concentrations compared to individuals without the disease [12].

Taurine plays protective cellular roles, including conjugation with bile acids to form bile salts. Primary bile acids such as cholic acid (CA) and chenodeoxycholic acid (CDCA) are synthesised in hepatocytes and conjugated with taurine by bile acid CoA‐ligase (BAL) and bile acid CoA:amino acid N‐acyltransferase (BAAT), generating taurine‐conjugated bile acids such as taurocholic acid (TCA) and taurochenodeoxycholic acid (TCDCA) [13]. Thus, taurine metabolism is closely linked to bile acid dynamics and gut microbiota activity.

Following dietary intake, taurine‐conjugated bile acids (Tau‐BAs) are secreted into the small intestine. There, microbial bile salt hydrolases (BSHs) deconjugate them, releasing free taurine. The resulting bile acids are subsequently converted by gut microbes into secondary bile acids, promoting faecal excretion [13].

Genes encoding BSH enzymes have been identified in the human gut microbiota and are distributed across the phyla Actinobacteria, Bacteroidetes, Firmicutes, and Proteobacteria. Notably, a substantial proportion of the identified BSHs is associated with bacteria from the phylum Firmicutes, including members of the genus *Clostridium* [14].

In the study by Li et al. [4], healthy individuals showed a higher relative abundance of Firmicutes than those with T1DM. Conversely, reduced Clostridia abundance was reported in individuals with long‐standing T1DM. Taken together with previous evidence regarding BSH‐producing bacteria and bile acid deconjugation [13, 14] it is plausible to hypothesise that reduced *Firmicutes* abundance might influence bile acid metabolism and, consequently, intestinal and systemic taurine availability. However, since BSH activity were not assessed in the study by Li et al. [4], this proposed BSH–taurine axis remains speculative and should be considered a mechanistic hypothesis that requires direct experimental validation.

Future studies integrating microbial profiling, bile acid metabolomics, and direct assessment of BSH activity are needed to establish whether the proposed interplay between gut microbiota, bile acid metabolism, T1DM pathogenesis, and taurine availability is biologically relevant. These investigations may determine whether modulation of BSH‐producing bacteria might represent a promising therapeutic strategy for restoring taurine homoeostasis, an amino acid that has attracted increasing attention in diabetes research.

A key strength of Li et al. [4] is the integrated analysis of gut microbiota and serum metabolome across T1DM durations, an underexplored area. However, small subgroup sizes may limit statistical power and generalisability. The cross‐sectional design also precludes causal inference, highlighting the need for longitudinal and mechanistic studies to determine whether microbial changes drive disease progression or reflect consequences of prolonged T1DM and metabolic dysregulation.

Gut microbiota is strongly influenced by diet and medication; thus, studies should control for key confounders, particularly diet, treatments, and insulin type. Overall, microbiota‐mediated taurine metabolism may represent a promising area for investigation in T1DM, particularly regarding its potential role in modulating immune and metabolic dysfunction.

## Author Contributions

C.F. conceptualization, writing. G.U.O. writing, review and editing. A.S.R.S. review and editing. E.C.F. review and editing, supervision.

## Funding

FAPESP, 2023/15008‐9, C.F.; FAPESP, 2022/00221‐6, G.U.O.; CNPq, 308999/2022‐3, A.S.R.S.; 303766/2022‐0, E.C.F.

## Ethics Statement

The authors have nothing to report.

## Conflicts of Interest

The authors declare no conflicts of interest.

## Data Availability

Data sharing not applicable to this article as no datasets were generated or analysed during the current study.
